# Nuclear Factor of Activated T Cells and Cytokines Gene Expression of the T Cells in AIDS Patients with Immune Reconstitution Inflammatory Syndrome during Highly Active Antiretroviral Therapy

**DOI:** 10.1155/2017/1754741

**Published:** 2017-02-20

**Authors:** Jia Sun, Heling Chen, Yirui Xie, Junwei Su, Ying Huang, Lijun Xu, Michael Yin, Qihui Zhou, Biao Zhu

**Affiliations:** ^1^State Key Laboratory for Diagnosis and Treatment of Infectious Diseases, Collaborative Innovation Center for Diagnosis and Treatment of Infectious Diseases, The First Affiliated Hospital, School of Medicine, Zhejiang University, Hangzhou 310003, China; ^2^Division of Infectious Diseases, Columbia University Medical Center, New York, NY 10032, USA

## Abstract

*Background*. The etiology of immune reconstitution inflammatory syndrome (IRIS) in AIDS patients after the initiation of HAART remains unknown. Several researches indicated that the development of IRIS is associated with the production and variation of cytokines, whose gene expression are closely related to the Ca2^+^/CN-nuclear factor of activated T cells (NFAT) pathway.* Methods*. We studied the expression of NFAT isoforms and their major target cytokines genes in peripheral blood CD3^+^ T cells of subjects through fluorescence quantitative PCR and explored the expression changes of these genes before and after HAART.* Results*. After the initiation of HARRT, NFAT1, IL-6, and IL-8 gene expression showed a reversal trend in the CD3^+^ T cells of the IRIS group and changed from low expression before HARRT to high expression after HARRT. In particular, the relative gene expression of NFAT1 was markedly higher compared with the other three isoforms. The IRIS group also showed higher NFAT4, NFAT2, NFAT1, IL-1*β*, IL-10, IL-2, IL-18, and TNF-*α* gene expression than the non-IRIS group.* Conclusion*. This study suggested that high expression levels of IL-2, IL-6, IL-8, TNF-*α*, IL-1*β*, IL-10, IL-12, and IL-18 can predict the risk of IRIS. The increased expression of NFAT1 and NFAT4 may promote the expression of cytokines, such as IL-6, IL-8, and TNF-*α*, which may promote the occurrence of IRIS.

## 1. Introduction

Highly active antiretroviral therapy (HAART) has substantially reduced the incidence and mortality of HIV-related diseases in AIDS patients. However, 8–40% of patients still have excessive inflammatory responses to various infectious or noninfectious pathogens, mostly within six months of HAART, leading to increased clinical symptoms. These patients present with aggregated opportunistic infections that have been effectively treated or with emerging infections, which is called immune reconstitution inflammatory syndrome (IRIS) [1–5]. IRIS is associated with various infectious diseases, and tuberculosis-related IRIS constitutes the highest incidence of IRIS at 8–43%; the incidence is even higher in developed countries (17–43%) [6]. Other infections associated with IRIS include* Mycobacterium avium* infection, cryptococcosis, pneumocystis pneumonia, and cytomegalovirus retinitis; noninfectious diseases include various autoimmune diseases such as Graves' disease and Kaposi's sarcoma [7].

IRIS is likely to occur in patients who have high viral loads and low CD4 cardinality (<50/*μ*L) before HAART, with a rapid decline in the viral load and a fast increase in CD4 counts after HAART. Additionally, IRIS easily occurs in patients who have a short interval between the treatment of opportunistic infections and the start of HAART. Moreover, IRIS has been reported to easily occur in patients with low baseline hemoglobin but high C-reactive protein and plasma D-dimer levels [5–10]. However, the pathogenesis of IRIS remains unclear, and there are great differences among the various types of IRIS. It has been reported that tuberculosis-associated IRIS (TB-IRIS) is associated with interleukin-4 (IL-4), IL-6, IL-7, IL-12p40, interferon-*γ* (IFN-*γ*), and tumor necrosis factor-*α* (TNF-*α*); an elevation in IL-12 can increase the incidence of cytomegalovirus-associated IRIS (CMV-IRIS), whereas IFN-*γ*, IL-12p40, IL-6, IL-4, IL-17, and TNF-*α* are relevant to* Cryptococcus neoformans*-associated IRIS (*C. neoformans*-IRIS) [11–13]. The nuclear factor of activated T cells (NFAT) is expressed on the promoter/enhancer of multiple genes mentioned above, including IL-2, IL-3, IL-4, IL-5, IL-6, IL-8, IL-10, IL-13, IFN-*γ*, TNF-*α*, and granulocyte-macrophage colony-stimulating factor (GM-CSF); NFAT regulates the expression of its target genes mainly through the Ca^2+^/CN-NFAT pathway [14–16].

To further elucidate the pathogenesis of IRIS, we analyzed gene expression changes of NFAT1–NFAT4 and the major target genes IL-2, IL-6, IL-8, TNF-*α*, IL-1*β*, IL-10, and IL-12 in peripheral blood CD3^+^ T cells before and during the course of IRIS.

## 2. Materials and Methods

### 2.1. Study Population and Baseline Profile

This study enrolled 63 AIDS patients at the Infectious Diseases Center of the First Affiliated Hospital, Zhejiang University School of Medicine (Hangzhou, Zhejiang Province, China), from March 2003 to December 2014, of which 19 subjects have been diagnosed with IRIS at the time they entered the study. Of the remaining 44 patients, 5 have initiated HAART for 2 weeks when they agreed to participate in the study, and 39 subjects have not. Under observation for as long as six months, 11 subjects had IRIS and 33 did not. Above all, there were 30 patients with IRIS (IRIS group: 28 males and 2 females) and 33 patients without IRIS (non-IRIS group: 30 males and 3 females). The IRIS group included 13 cases with TB-IRIS, five cases with CMV-IRIS, three cases with* C. neoformans*-IRIS, five cases with varicella zoster virus-associated IRIS, two cases with pneumocystis pneumonia-associated IRIS, one case with cytomegalovirus/tuberculosis coinfected IRIS, and one case with* Penicillium marneffei-*associated IRIS. IRIS was diagnosed according to the criteria of the International Network for the Study of HIV-Associated IRIS. The baseline conditions of the subjects are detailed in Table 1. This study was approved by the hospital ethics committee, and each patient who was enrolled had signed an informed consent form.

### 2.2. Specimen Collection and Processing

Whole blood specimens (5–8 mL each) were collected from the all subjects at the initiation and 3 months after starting HAART. Moreover, for the IRIS group, blood specimens were collected at the diagnosis time point of IRIS and 1 month after HAART for the non-IRIS group. The specimens were anticoagulated with EDTA. Plasma and PBMCs were isolated within 10 hours of collection. The PBMCs were stored in Zenoaq's CELLBANKER cryopreservation medium at −80°C. We examined gene expression changes in the IRIS and non-IRIS groups before and 1 month after HAART.

### 2.3. Detection of NFAT and the Expression of Its Target Genes in T Cells

#### 2.3.1. Magnetic Bead Separation of CD3^+^ T Cells and RNA Extraction of Samples

The PBMCs were thawed in a warm bath at 37°C and centrifuged at 300 g/min for 5 min. The supernatant was aspirated, and the CD3^+^ T cells were isolated using CD3 human MicroBeads (Miltenyi Biotec, Bergisch Gladbach, Germany) by strictly following the manufacturer's instructions. The isolated CD3^+^ T cells were added to 1 mL of TRIzol reagent (Invitrogen, Life Technologies, Carlsbad, CA, USA), and total RNA extraction was performed according to the manufacturer's instructions. The extracted RNA was dissolved with 20 *μ*L of DEPC water before the RNA concentration measurement.

#### 2.3.2. cDNA Synthesis

The cDNA synthesis reactions were prepared on ice using a PrimeScript RT reagent kit (Takara, Dalian, China). The 40 *μ*L reaction contained 8 *μ*L of 5x PrimeScript Buffer (for real time), 2 *μ*L of PrimeScriptRT Enzyme Mix I, 2 *μ*L of Oligo dT Primer (50 *μ*M), 2 *μ*L of random 6 mers (100 *μ*M), 500 ng of total RNA, and up to 40 *μ*L of RNase-free dH_2_O. The reaction conditions were as follows: 37°C for 45 min, 85°C for 5 sec, and 4°C for 1 hour. The synthesized cDNA was stored at −20°C.

#### 2.3.3. Fluorescence Quantitative RT-PCR

We performed qPCR reactions using iQ^™^ SYBR Green Supermix (Bio-Rad, Hercules, CA, USA). The reaction system contained 12.5 *μ*L of Bio-Rad iQ SYBR Green Supermix, 1 *μ*L of sense primer (50 pg), 1 *μ*L of antisense primer (5 pg), 1 *μ*L of cDNA, and 9.5 *μ*L of DEPC H_2_O, resulting in a total volume of 25 *μ*L. The reaction conditions were as follows: 95°C for 3 min, followed by 45 cycles of 95°C for 15 sec, 60°C for 30 sec, and 72°C for 30 sec. A melting curve analysis was performed using the following conditions: 65°C to 95°C for 5 s. The primer sequences are shown in Table 2. The primers were synthesized by Sangon (Shanghai, China). The PCR reactions were performed on a quantitative PCR machine (Bio-Rad).

## 3. Statistical Methods

All of the data were analyzed using IBM SPSS Statistics 19 (IBM SPSS, Somers, NY, USA) unless otherwise stated. We first performed a one-sample K-S test to examine the normal distribution of the data. Then, a *T*-test was used to analyze the normally distributed data, and the Mann–Whitney *U* test was used to analyze the nonnormally distributed data. Differences were considered statistically significant at *p* < 0.05. All of the graphs were generated using GraphPad Prism 5 (GraphPad Software, Inc., San Diego, CA, USA). We compared previous gene expression changes to 1 month after HAART in the IRIS and non-IRIS groups, respectively. Meanwhile, we compare IRIS group to non-IRIS group at the time before HAART and 1 month after HAART separately by using the fold difference (2^−ΔΔCT^) method, with human GAPDH as an internal reference. The Delta-Delta-Threshold cycle (ΔΔCT) equation was used to compare the expressions of NFAT and cytokine genes in IRIS and non-IRIS patients before and after HAART in terms of fold difference according to the following formulas:

  ΔCT = CT value of targeted gene − CT value of GAPDH.
  ΔΔCT = [average ΔCT value of the IRIS group − average ΔCT value of the non-IRIS group] or [average ΔCT value of patients before HAART − average ΔCT value of patients after HAART].
  Fold difference = [2]^−ΔΔCT^.

## 4. Results

### 4.1. NFAT and Cytokine Gene Relative Expression Levels Compared before and after HAART in the IRIS and Non-IRIS Groups of AIDS Patients

**Figure 1:**
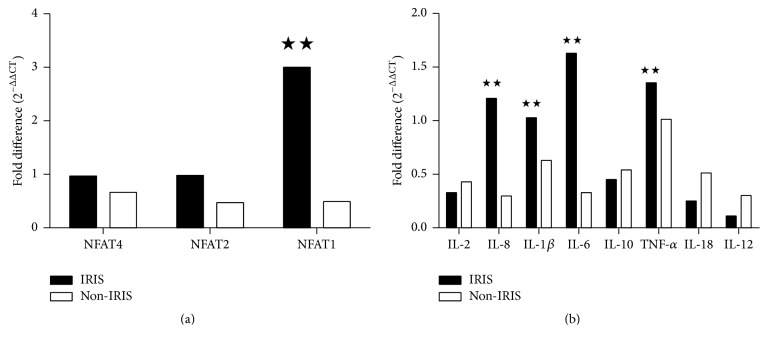
NFAT and cytokine gene relative expression levels compared before and after HAART in the immune reconstitution inflammatory syndrome (IRIS) and non-IRIS groups of AIDS patients. (a) NFAT expression in IRIS groups before and after HAART; NFAT1 expression from low to high after HAART; the relative expression of mRNA for NFAT1 gene to GAPDH after HAART was 3.002-fold higher as compared to that before HAART. (b) Cytokine gene expression in IRIS groups before and after HAART; IL-1*β*, IL-6, IL-8, and TNF-*α* expression from low to high; TNF-*α* gene expression also increased in non-IRIS group after HAART;  ^★★^the gene expressions after HAART were higher than those before treatment.

A comparative analysis of the relative gene expression by fold difference before and after HAART revealed that the IRIS group had higher NFAT1, IL-6, IL-8, and TNF-*α* expression in CD3^+^ T cells after receiving HAART for 1 month. The gene expression increase was the most obvious for NFAT1, whose expression after HAART was 3.058-fold greater than that before HAART. However, gene expression of IL-2, IL-10, and IL-18 was lower after HAART. The differences of gene expressions of NFAT4, NFAT2, and IL-1*β* were not obvious after initiation of HAART (fold difference: 0.969, 0.981, and 1.03, resp.). The non-IRIS group showed lower NFAT4, NFAT2, NFAT1, IL-1*β*, IL-6, IL-8, IL-10, IL-2, and IL-18 gene expressions after HAART than before HAART. The gene expression of TNF-*α* after HAART changed little, and it was 1.03-fold higher than that before HAART (Figure 1 and Table 3).

### 4.2. NFAT and Cytokine Gene Relative Expression Levels Comparing the IRIS to Non-IRIS Groups of AIDS Patients before and after HAART

**Figure 2:**
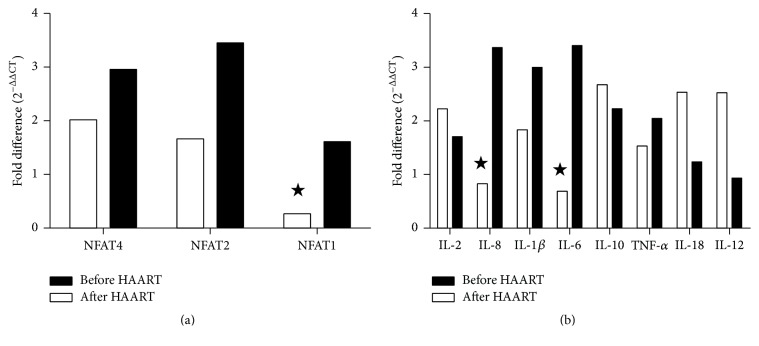
NFAT and cytokine gene relative expression levels comparing the IRIS to non-IRIS groups of AIDS patients before and after HAART. (a) NFAT expression in IRIS and non-IRIS groups before HAART. NFAT1 expression was obviously lower in IRIS group. (b) Cytokine gene expression in IRIS and non-IRIS groups before HAART. IL-6 and IL-8 expressions were obviously lower in IRIS group;  ^★^before HAART, the gene expressions in IRIS group were lower than those in non-IRIS group.

We compared the relative gene expression levels of NFAT and cytokines between the IRIS and non-IRIS groups before HAART. NFAT1 was expressed at significantly lower levels than the other three NFAT isoforms in the CD3^+^ T cells of the IRIS group. The IRIS group showed lower IL-6 and IL-8 expression levels than the non-IRIS group, whereas other genes including NFAT4, NFAT3, NFAT2, IL-1*β*, IL-10, IL-2, IL-18, and TNF-*α* were expressed at higher levels in the IRIS group than in the non-IRIS group. After 1 month of HAART, NFAT1, IL-6, and IL-8 expressions showed a reversal trend and changed from low expression levels before HAART to high expression levels after HAART. The relative expressions of NFAT1, IL-6, and IL-8 genes in the IRIS group were 1.61-, 3.406-, and 3.368-fold greater than those in the non-IRIS group, respectively; other genes like NFAT4, NFAT2, IL-1*β*, IL-2, IL-10, TNF-*α*, and IL-18 were also expressed at higher levels compared with those in the non-IRIS group. However, the relative expressions of IL-10, IL-2, and IL-18 showed a larger decreasing amount than those in the non-IRIS group (Figure 2 and Table 3).

## 5. Discussion

IRIS easily occurs in patients who have a short interval between the treatment of opportunistic infections and the start of HAART [5]. As a result, the occurrence of IRIS was related with residual antigen burden. Taken the active tuberculosis as an example, before antituberculosis therapy, there existed high level of TB antigen, so the first step for TB AIDS patients was anti-TB therapy and then HAART, which was similar in the therapy of CMV AIDS. Generally speaking, the development of IRIS associated with pathogen infection; therefore the major principle of IRIS treatment is the antipathogen therapy for a period of time before the initiation of HAART, through which it can not only effectively decrease the antigen burden but also cut down the scale of residual antigen. Meanwhile, to reduce the occurrence of IRIS, methods like T-SPOT, CMV-DNA, and VZV-DNA are widely used to measure the antigen burden and residual antigen in clinic.

The pathogenesis of IRIS has not been clarified yet. Tadokera et al. found that IL-1*β*, IL-5, IL-6, IL-10, IL-13, IL-17A, IFN-*γ*, GM-CSF, and TNF levels are elevated in the cell culture of PBMCs from TB-IRIS patients after in vitro stimulation with inactivated* Mycobacterium tuberculosis*. This finding indicates that the release of cytokines promotes the occurrence of TB-IRIS [17]. The cytokine release syndrome (also known as the cytokine storm) can be observed in infectious or noninfectious diseases, such as graft-versus-host disease and acute respiratory distress syndrome [17]. Currently, calcineurin inhibitors including cyclosporine (CSA) and tacrolimus (FK506) are available to block the Ca^2+^-CN/NFAT pathway and suppress the function and spread of effector T cells. These agents have been extensively used in transplantation patients. Moreover, calcineurin inhibitors can inhibit the formation of IL-2, IL-4, and TNF-*α* [16]. Therefore, we speculate that the Ca^2+^-CN/NFAT pathway may play a role in the excessive immune response of AIDS patients receiving HAART.

We performed fluorescence quantitative PCR analysis to detect NFAT1–NFAT4, IL-2, IL-6, IL-8, TNF-*α*, IL-1*β*, IL-10, and IL-12 genes in the CD3^+^ T cells of IRIS and non-IRIS patients. The results showed that the relative gene expression of NFAT1, IL-6, IL-8, and TNF-*α* was obviously higher after 1 month of HAART than before HAART in the CD3^+^ T cells of IRIS patients and that the most significant gene expression increase was obtained for NFAT1. When the IRIS group was compared to the non-IRIS group, we found that, before HAART, IL-1*β*, IL-10, and IL-2 expressions of CD3^+^ T cells were higher in the former than in the latter group; after 1 month of HAART, NFAT2, NFAT4, IL-6, IL-8, TNF-*α*, IL-1*β*, IL-10, and IL-2 expression levels were all higher in the former than in the latter group. IL-1*β*, IL-10, and IL-2 expressions were reduced in both the IRIS and non-IRIS groups after HAART; the reduction was larger in the non-IRIS group than in the IRIS group. Maintaining relatively high expression levels of IL-1*β*, IL-10, and IL-2 may play an important role in the development and progression of IRIS. The increase in NFAT1 expression in IRIS patients after 1 month of HAART likely plays an essential role in the occurrence of IRIS by contributing to IL-6, IL-8, and TNF-*α* expression,

NFAT can bind to the promoter/enhancer of various cytokine genes and can contribute to the expression of IL-2, IL-3, IL-4, IL-5, IL-6, IL-8, IL-10, IL-13, IFN-*γ*, and TNF-*α*. During HAART for AIDS, we found NFAT expression changes in the T lymphocytes of the IRIS patients. Among four NFAT1 isoforms, NFAT1 and NFAT4 showed an increase in expression; the expression increase was more significant for NFAT1. These results provided a new idea for the treatment of IRIS: we can use selective inhibitors of iRNA or NFAT1 to block the CN/NFAT pathway and reduce IL-2, IL-3, IL-4, IL-5, IL-6, IL-8, IL-10, IL-13, IFN-*γ*, and TNF-*α* generation for the treatment of IRIS to reduce the occurrence of IRIS and improve the prognosis of IRIS patients in the clinic.

Taken powerful immunosuppressive action of calcineurin inhibitors, such as CSA and FK506, into consideration, we replace thalidomide, which is an anti-inflammatory, immunomodulatory, and antiangiogenic agent [18] and has already been effectively used in our recent case report of* Mycobacterium avium complex-* (MAC-) associated IRIS, whose cutaneous MAC lesions continue exacerbation despite antimycobacterial treatment. But rapid clinical remission occurred after adding thalidomide to the antituberculosis treatment. Furthermore, immunomodulatory effect of thalidomide on cytokine levels such as decreasing of TNF-*α* and increasing of IL-4 and IL-6 was observed in the patient [19]. The limitation for this approach of blocking the CN/NFT pathway is that this inhibition could negatively affect immune responses activation when these patients have real/new infections, as the immune system is still under severely immune suppression. Anyway, the development of IRIS also indicated that these patients have an excellent effect in response to HAART. As it is a condition of immunoimbalance, under the written informed consents from all subjects, we are conducting a case-control study by using CN/NFT drugs from small doses in study group and observe the effects intensively.

## Figures and Tables

**Table 1:** General information of the 63 AIDS patients enrolled in this study.

	IRIS (30 cases)	Non-IRIS (33 cases)	*p*
*Gender*			
Male	28	30	>0.05
Female	2	3	>0.05
*Average age*	40.5	35.8	>0.05
*CD4* ^*+*^ * T (cells/mm* ^*3*^)			
Pretreatment	45	150	<0.001
1-Month treatment	135	295	>0.05
*HIV-RNA (log 10 copies/mL)*			
Pretreatment	5.52	5.627	>0.05
1-Month treatment	4.22	5.36	>0.05
*Hemoglobin (g/dL)*			
Pretreatment *Mean duration of IRIS (days)*	11.5 31	13.6 /	>0.05
*Therapeutic strategy*			
2NRTI + NNRTI	27	30	>0.05
2NRTI + PI	3	3	>0.05

NRTI: nucleoside reverse transcriptase inhibitors; NNRTI: nonnucleoside reverse transcriptase inhibitors; PI: protease inhibitor.

**Table 2:** Primer sequences.

Gene	Primer sequence	Product length (bp)
IL-2	F: TGCATTGCACTAAGTCTTGCAC	194
	R: AGTTCTGTGGCCTTCTTGGG	
TNF-*α*	F: CCCTGGTATGAGCCCATCTAT	298
	R: CGAAGTGGTGGTCTTGTTGC	
IL-8	F: TGAATGGGTTTGCTAGAATGTG	223
	R: CTGTGAGGTAAGATGGTGGCTAA	
IL-6	F: AGTAGTGAGGAACAAGCCAGAG	240
	R: ATGCTACATTTGCCGAAGAG	
IL-10	F: GCCCCTTGAGAAACCTTATTGT	115
	R: GGCTTCTTTCTAAATCGTTCACAG	
IL-1*β*	F: GCACCTCTCAAGCAGAAAACA	207
	R: ACAACAGGAAAGTCCAGGCTAT	
IL-18	F: GCTGAAGATGATGAAAACCTGGA	118
	R: GAGGCCGATTTCCTTGGTCA	
IL-12A	F: TTCACCACTCCCAAAACCTGC	226
	R: GAGGCCAGGCAACTCCCATTAG	
NFAT1	F: AAGAGCCAGCCCAACATGC	107
	R: CGTTTTCTCTTCCCATTGATGAC	
NFAT2	F: CTGTGCAAGCCGAATTCTCTGG	78
	R: ACTGACGTGAACGGGGCTGG	
NFAT4	F: GCGGCCTGCAGATCTTGAGC	102
	R: TGATGTGGTAAGCAAAGTGGTGTGGT	
GAPDH	F: GGCCTCCAAGGAGTAAGACC	147
	R: AGGGGTCTACATGGAAACTG	

**Table 3:** Fold change expression and quantitative PCR results of NFAT and cytokines genes.

	Fold difference 1	Fold difference 2	Fold difference 3	Fold difference 4
NFAT4	0.661	0.969	2.017	2.957
NFAT2	0.473	0.983	1.661	3.452
NFAT1	0.493	3.002	0.265	1.612
IL-2	0.428	0.329	2.223	1.707
IL-8	0.298	1.210	0.829	3.368
IL-1*β*	0.629	1.027	1.836	3.000
IL-6	0.329	1.629	0.687	3.406
IL-10	0.541	0.451	2.674	2.229
TNF-*α*	1.013	1.335	1.533	2.050
IL-18	0.512	0.250	2.536	1.239
IL-12	0.301	0.111	2.525	0.934

Fold  difference = [2]^−ΔΔCT^.

ΔΔCT1 = [average ΔCT value of non-IRIS group after HAART − average ΔCT value of non-IRIS group before HAART].

ΔΔCT2 = [average ΔCT value of IRIS group after HAART − average ΔCT value of IRIS group before HAART].

ΔΔCT3 = [average ΔCT value of IRIS group before HAART − average ΔCT value of non-IRIS group before HAART].

ΔΔCT4 = [average ΔCT value of IRIS group after HAART − average ΔCT value of non-IRIS group after HAART].
